# Reference genome for the benthic marine diatom *Psammoneis japonica*: Bacterial associations and repeat‐driven genome size evolution in diatoms

**DOI:** 10.1111/jpy.70101

**Published:** 2025-11-10

**Authors:** Wade R. Roberts, Matthew Parks, Marissa Ashner, Matthew P. Ashworth, Nina Denne, Elizabeth C. Ruck, Elias Spiliotopoulos, Anni Wang, Shady A. Amin, Sarah Schaack, Norman J. Wickett, Andrew J. Alverson

**Affiliations:** ^1^ Department of Biological Sciences University of Arkansas Fayetteville Arkansas USA; ^2^ Department of Biology University of Central Oklahoma Edmond Oklahoma USA; ^3^ Department of Applied Mathematics Illinois Institute of Technology Chicago Illinois USA; ^4^ Department of Molecular Biosciences University of Texas at Austin Austin Texas USA; ^5^ Biology Department Carleton College Northfield Minnesota USA; ^6^ Biology Department Reed College Portland Oregon USA; ^7^ Department of Biological Science Florida State University Tallahassee Florida USA; ^8^ Biology Program New York University Abu Dhabi Abu Dhabi United Arab Emirates; ^9^ Department of Botany and Biodiversity Research University of Vienna Vienna Austria

**Keywords:** araphid, diatoms, metagenome, phycosphere, *Psammoneis*

## Abstract

We sequenced the genome, transcriptome, and bacterial metagenome of *Psammoneis japonica*, a benthic, chain‐forming, and araphid marine diatom. This combination of traits fills several gaps in genome sequencing coverage across diatoms. The nuclear genome (QPGO00000000) is an estimated 91.4 Mb in length, with 11,047 genes that comprise 18% of the total genome. Repetitive elements account for 33% of the genome, and other noncoding sequences comprise the remaining 49% of the genome. A global analysis of diatom genomes showed that repetitive elements are the principal driver of genome size variation in diatoms. Four complete genomes of Planctomycetota, ɑ‐proteobacteria, and Bacteroidota were also recovered, and each had only moderate similarity to previously sequenced bacterial genomes. This finding supports the idea that bacterial species richness in the phycosphere is under‐described and far exceeds the number of diatom host species, which themselves number in the tens to hundreds of thousands of species.

**Abbreviations**
aLRT: approximate likelihood ratio test
ANI: average nucleotide identity
LTR: long terminal repeat
NCBI: National Center for Biotechnology Information
PGLS: phylogenetic generalized least squares
SEM: scanning electron microscope
SMRT: single‐molecule, real‐time sequencing

## INTRODUCTION

Considerable progress has been made in the field of diatom genomics since publication of the first diatom genome in 2004 (Armbrust et al., 2004). Draft‐ or reference‐quality genomes have been sequenced for dozens of species (Nenasheva et al., 2025; Roberts et al., 2024), and transcriptomes are available for hundreds more (Alverson et al., 2025; Keeling et al., 2014). Individual genomes have revealed previously undescribed metabolic pathways (Armbrust et al., 2004; Onyshchenko et al., 2021), provided insights into cellular motility (Osuna‐Cruz et al., 2020), and shown environmentally driven acquisitions of foreign genes (Lim et al., 2025). Comparative studies meanwhile have revealed differences in gene number and content (Nenasheva et al., 2025), a deep history of genome duplication (Parks et al., 2018), and genome sizes that range from tens to many hundreds of megabases (Di Costanzo et al., 2025; Roberts et al., 2024). Genome size is an especially important trait due to its cascading effects on cell size, growth rate, and community abundance (Connolly et al., 2008; Roberts et al., 2024; Sharpe et al., 2012). Broad sampling of diatom genomes provides opportunities to test these and other hypotheses about the causes and mechanisms of genome size evolution, such as the roles of repetitive elements and polyploidy (Hermann et al., 2014; Maumus et al., 2009; Parks et al., 2018).

Diatoms and other algae are surrounded by a consortium of bacteria that exist in a diffusive boundary layer surrounding the cell known as the phycosphere (Bell & Mitchell, 1972; Seymour et al., 2017). These bacteria can promote algal growth in a variety of ways, including through the production of growth‐enhancing hormones (Amin et al., 2015; Segev et al., 2016), through the provision of the essential B_12_ vitamin (Bell & Mitchell, 1972, Seymour et al., 2017), or by mediating the assimilation of growth‐limiting iron (Amin et al., 2009). Although some diatoms can be cultured in the absence of bacteria under nutrient‐rich conditions, many species cannot, presumably due to the essential functions provided by their bacterial partners. The diatom *Pseudo‐nitzschia subcurvata*, for example, grows poorly or not at all when grown in vitamin‐deficient, axenic conditions (Andrew et al., 2022). As a result, many diatom genome sequencing projects are metagenomic in nature, producing DNA from both the diatom and its cohabitating bacteria. The bacterial components, however, are often unreported, excluding potentially valuable insights into the cellular physiology and ecology of the diatom.

We have expanded the phylogenetic and ecological diversity of sequenced diatom genomes through sequencing and analysis of the genome of *Psammoneis japonica*, a benthic marine diatom that forms cell colonies. Comparative genomic analyses revealed key factors driving the evolution of genome size and content in diatoms. We also recovered the partial metagenome of the *P. japonica* microbiome by co‐sequencing the complete genomes of four cohabitating bacterial species, all of which are new to science.

## MATERIALS AND METHODS

### Culturing, DNA extraction, and sequencing

*Psammoneis japonica* strain UTEX LB 3220 (synonym ECT2AJA‐110) was isolated from a collection made in 2014 at Outhouse Beach, Guam (13.464200° N, 144.655000° E). The strain was maintained under 12:12 h light:dark conditions at 23°C in L1 medium (Guillard & Hargraves, 1993). The culture is available through the UTEX Culture Collection of Algae at The University of Texas at Austin.

Cells were concentrated by centrifugation and disrupted with a Mini‐Beadbeater (Biospec Products), and DNA was extracted using the DNeasy Plant Mini Kit (Qiagen). One genomic library was constructed using the TruSeq DNA kit (Illumina) and sequenced on the Illumina HiSeq2000 platform with 100 bp paired‐end reads. Another library was constructed using SMRTbell™ Library preparation (PacBio) with BluePippin size selection for single‐molecule, real‐time sequencing (SMRT) cell sequencing on the PacBio RSII platform. Both library preparation and sequencing were performed at the University of Delaware DNA Sequencing and Genotyping Center.

### RNA extraction and sequencing

Cells were harvested during exponential phase growth and concentrated by centrifugation. Total RNA was extracted from bead‐disrupted cells with a Qiagen RNeasy Kit (Qiagen), including DNase treatment. A single library was constructed using the TruSeq RNA Sample Preparation Kit v2 (Illumina), and 100 bp paired‐end reads were sequenced using the Illumina HiSeq2000 sequencing platform. Raw RNA sequencing reads were cleaned and filtered as described in Alverson et al. (2025) to remove adapter sequences, common sequencing vectors, organellar reads, and rRNA sequences.

### Genome assembly

The haploid genome size of *Psammoneis japonica* was estimated with GenomeScope2 (Ranallo‐Benavidez et al., 2020) using a histogram of kmer counts (*k* = 31) calculated with Jellyfish (v.2.3.0; Marçais & Kingsford, 2011). Errors in the raw Illumina reads were corrected with ACE (Sheikhizadeh & Ridder, 2015), specifying an estimated genome size of 90 Mb. Reads were then quality‐trimmed with Trimmomatic (v.0.32; ILLUMINACLIP:TruSeq_adapters.fa:2:30:10 LEADING:10 TRAILING:10 SLIDINGWINDOW:4:15 MINLEN:80; Bolger et al., 2014). A preliminary assembly of the Illumina reads was performed with Ray (v.2.3.1; Boisvert et al., 2010) using a kmer value of 31. Assembly quality and contamination were assessed with Blobtools (v.1.1.1; Laetsch & Blaxter, 2017). Subsequently, reads putatively assigned to the organelles and bacteria were removed based on GC content, read depth, and taxonomic assignment. Retained reads were then reassembled using Ray, this time using a kmer size of 67. These contigs were used as pseudoreads in the PacBio assembly.

We assembled the long PacBio sequencing reads and Illumina contigs with Falcon (v.0.4.0; https://github.com/PacificBiosciences/FALCON), specifying a read length cut‐off of 7000, a minimum coverage of 3, and a maximum coverage depth and difference of 100. This assembly was assessed for contamination using Blobtools and putative contaminants were removed as described above. At this point, four contigs representing whole bacterial genomes were removed for analysis (see below). Retained reads were reassembled with Falcon, and this penultimate assembly was subjected to another round of bacterial read filtering with Blobtools.

Two additional strategies were employed to identify and separate prokaryotic and eukaryotic contigs. First, the filtered Illumina reads were aligned to the assembly with BWA MEM (v.0.7.12; Li, 2013), and an empirically determined cut‐off of 3× coverage was used to discriminate between endogenous (>3×) and exogenous (<3×) contigs. Second, we used an estimate of gene density on each contig to remove contaminants, since prokaryotic contigs should have higher gene densities than eukaryotic contigs (Mira et al., 2001). We translated each contig into all six reading frames with EMBOSS (v.6.6.0; Rice et al., 2000) and searched the translated proteins against the SwissProt database with the National Center for Biotechnology Information (NCBI) BLASTP (Camacho et al., 2009). Translated proteins with significant hits (e‐value ≤1e^−3^) were considered proxies for genes. We calculated gene density for each contig as the number of unique translated proteins with ≥1 hit to SwissProt per 1 Mb of contig length. Altogether, the final determination of contig origin was based on contig length, GC content, sequencing depth, BLAST hits to reference databases, and gene density. These criteria combined to give robust estimates of contig origin.

The final assembly was error‐corrected with Quiver (v.2.1.0; Chin et al., 2013) and Pilon (v.1.2.1; Walker et al., 2014), using the PacBio and Illumina reads, respectively. The consensus contigs were checked for possible misassemblies with REAPR (v.1.0.18; Hunt et al., 2013), and gaps were filled with GapCloser (v.1.12; Luo et al., 2012) using the Illumina reads. We assessed the quality and completeness of the final assembly with QUAST (v.5.0.0; Gurevich et al., 2013), BUSCO (v.5.1.3; ‐m geno; eukaryota_odb10 and stramenopiles_odb10 data sets; Simão et al., 2015), and Merqury (v.1.3; Rhie et al., 2020).

### Repeat element identification

Repetitive sequences were identified with RepeatModeler2 (v.2.0.5; Flynn et al., 2020), including the additional long terminal repeat (LTR) structural discovery pipeline (‐LTRstruct). The resulting repeat library was used to softmask repetitive sequences in the genome with RepeatMasker (v.4.1.5; ‐s ‐a ‐gff ‐norna ‐xsmall; https://www.repeatmasker.org/).

### Gene model prediction and annotation

The softmasked genome assembly was used for gene prediction with BRAKER3 (v.3.0.8; Gabriel et al., 2024). For transcript and protein evidence, we provided the filtered RNA reads and the Stramenopile partition of the OrthoDB protein database (v.12; Tegenfeldt et al., 2025). For the protein evidence, we replaced the predicted proteins from older annotations of *Cyclotella nana* (Thaps3) and *Phaeodactylum tricornutum* (Phatr2) with more recent versions (Thaps4 and Phatr3; Rastogi et al., 2018, Filloramo et al., 2021). BRAKER3 gene models were assessed for completeness with BUSCO (‐m prot; eukaryota_odb10 and stramenopiles_odb10 data sets) and OMArk (v.0.3.0; LUCA.h5 database; Nevers et al., 2024). Single‐exon gene overprediction can be an issue for newly sequenced genomes, so we also calculated the ratio of monoexonic:multiexonic genes using gFACs (v.1.1.2; Caballero & Wegrzyn, 2019) as suggested by Vuruputoor et al. (2023).

We searched the predicted proteome for protein domains with InterProScan (v.5.36–75.0; ‐iprlookup ‐dp ‐goterm; Jones et al., 2014) against the Pfam (v.32.0; El‐Gebali et al., 2019) and PANTHER (v.14.1; Mi et al., 2019) databases. The SwissProt (release 2024_06) database was searched with NCBI BLASTP (‐evalue 1e‐10 ‐num_alignments 1 ‐seg yes ‐soft_masking true ‐lcase_masking ‐max_hsps 1), and the UniProt Reference Proteomes database (release 2020_04) was searched using Diamond BLASTP (v.2.1.9; ‐‐evalue 1e‐10 ‐‐max‐target‐seqs 1 ‐‐sensitive ‐‐max‐hsps 1; Buchfink et al., 2015).

### Metagenome assembly, bacterial genomes, and taxonomic assignment

Four complete, circular‐mapping bacterial genomes were identified from the preliminary Falcon assembly and polished using the same procedure as for the *Psammoneis japonica* nuclear genome assembly. We used CheckM2 (Chklovski et al., 2023) quality analysis to check bacterial genomes for completeness and contamination. Bacterial genomes were annotated with Prokka (v.1.14.6; Seemann, 2014) and taxonomically classified with GTDB‐Tk (v.2.3.2; reference database r214; Chaumeil et al., 2022).

### Comparative genome data set

Diatom genome assemblies were downloaded from NCBI (last accessed May 26, 2025) or PhycoCosm (Grigoriev et al., 2021). The assembly for the bolidophyte species *Triparma laevis* f. *inornata* (Ban et al., 2023) was included as an outgroup. Assemblies with ≥80% completeness, measured with BUSCO (stramenopiles_odb10 data set), were included in subsequent analyses. For each genome, the total number of genes and summed gene length were calculated. To estimate the amount of repetitive DNA in each genome assembly, we ran RepeatModeler2 and masked the repeats using RepeatMasker. For each genome, we tallied the total amount of repetitive DNA and the amounts of different tandem and interspersed repeat classes.

### Species phylogeny

For each genome assembly in the comparative data set, we extracted the protein sequences of the identified BUSCO orthologs. For each ortholog, the protein sequences were aligned with MUSCLE (v.5.3; Edgar, 2021) and then trimmed with ClipKIT (v.2.4.1; −m smart‐gap; Steenwyk et al., 2020). We estimated a phylogenetic tree using the concatenated alignment with IQ‐TREE (v.2.4.0; ‐m LG + G ‐alrt 10,000 ‐‐runs 10; Minh et al., 2020). The phylogenetic tree was rooted on *Triparma* and ultrametricized using the chronos function in the R package APE (v.5.8) (Paradis et al., 2004).

### Predictors of genome size

We tested whether assembly size (≈genome size) was predicted by the total combined length of repetitive DNA, total combined gene length, or number of genes. We ran phylogenetic generalized least squares (PGLS) analyses on the data set of 42 diatom species using the pgls function in the R package caper (v.1.0.3; Orme et al., 2023), using maximum likelihood and setting the correlation structure to Pagel's lambda (Pagel, 1999). All variables were log‐10 transformed for these analyses.

## RESULTS AND DISCUSSION

**FIGURE 1:**
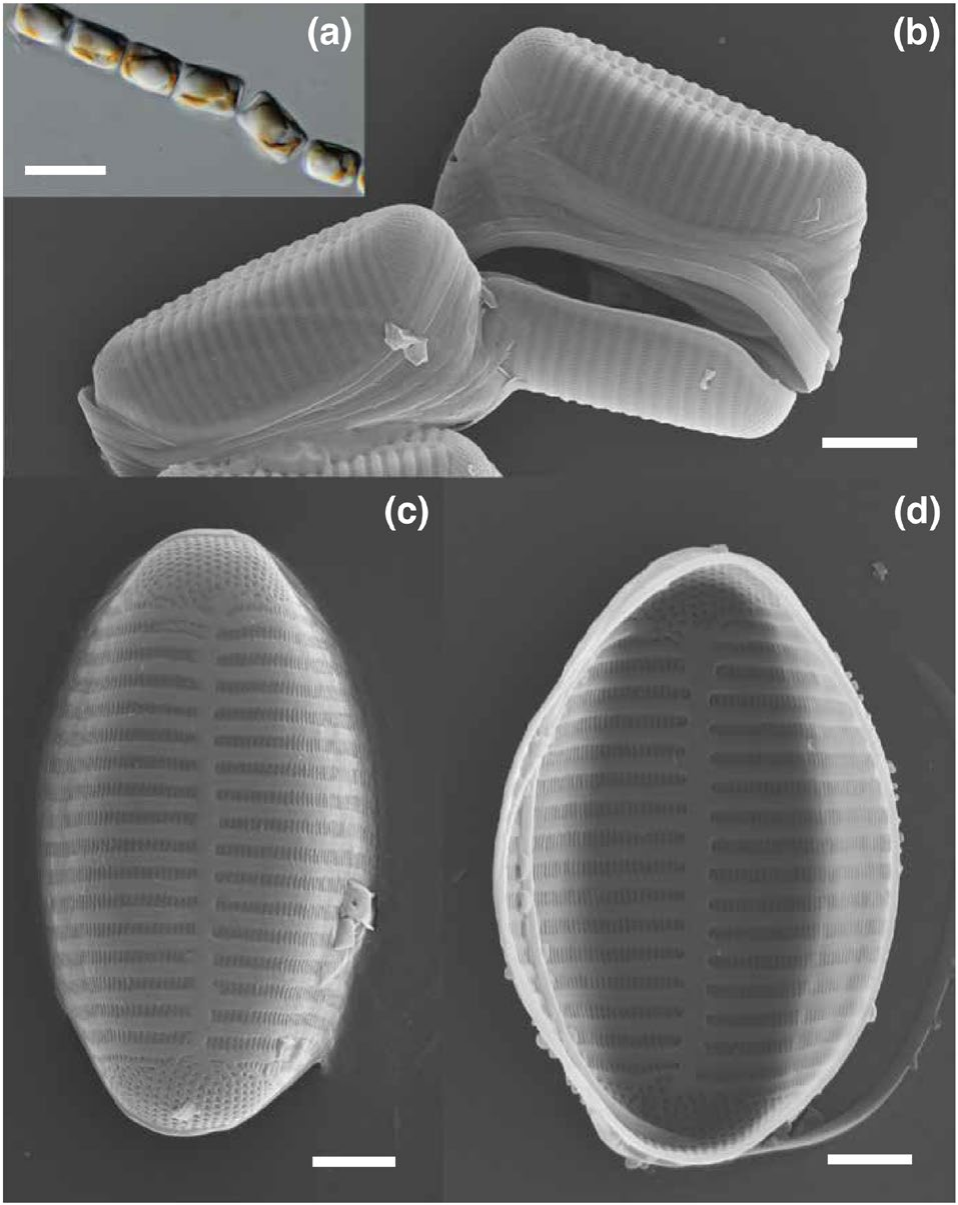
Light and scanning electron microscope (SEM) images of *Psammoneis japonica* strain UTEX LB 3220 (ECT2AJA‐110). (a) Light micrographs of live cells forming a chain (scale bar = 10 μm). (b–d) SEM micrographs show the cell exterior inside/girdle view (b; scale bar = 2 μm) and top/valve view (c; scale bar = 1 μm). Panel (d) shows the interior in valve view (scale bar = 1 μm).

**FIGURE 2:**
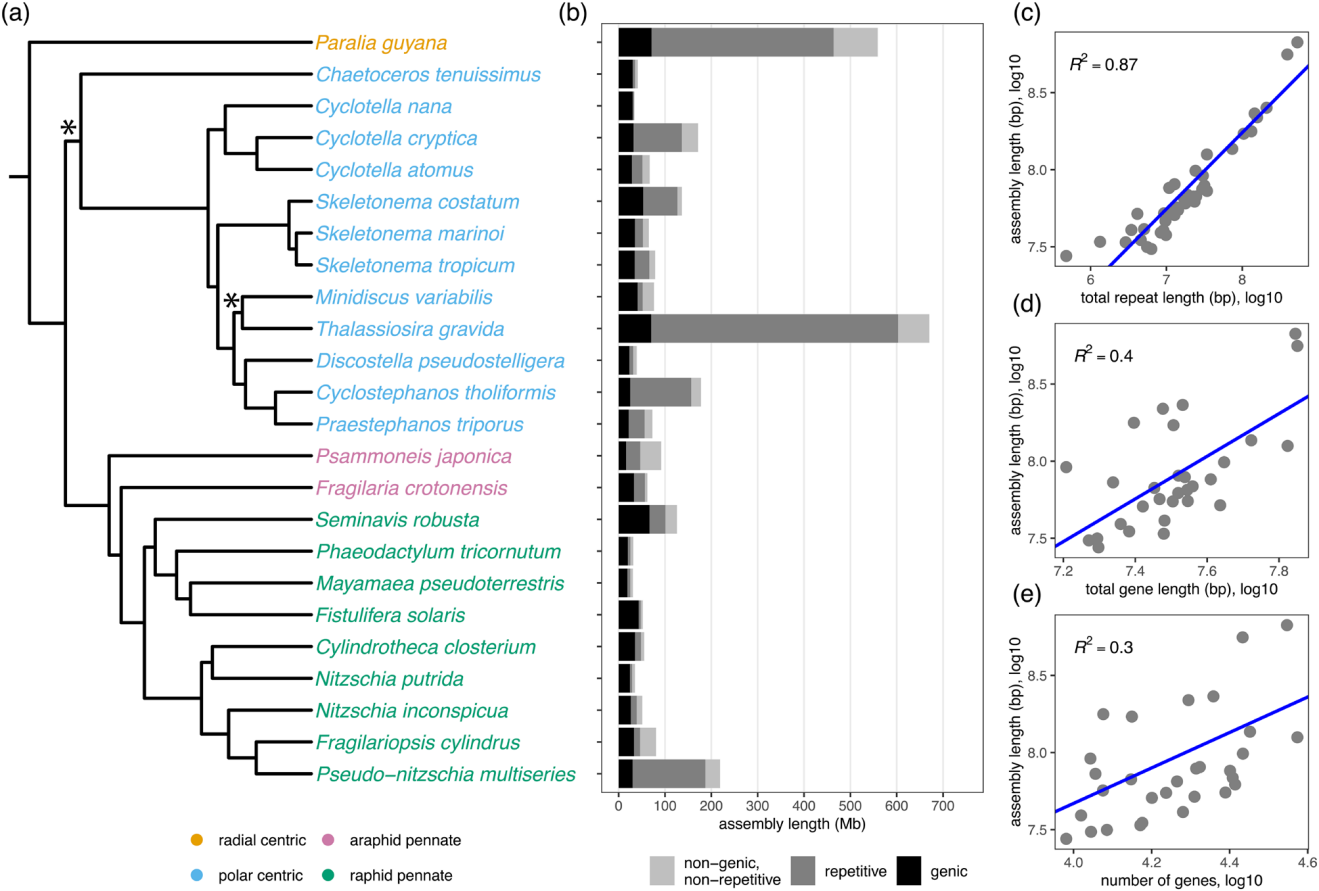
Genome size varies widely across diatoms. (a) Maximum likelihood phylogenetic analysis of high‐quality, long read‐based diatom reference genomes using 100 concatenated single‐copy orthologs. Nearly all branches had maximum support based on 10,000 aLRT replicates. The two branches with an asterisk had support less than 80. (b) The proportion of each genome assembly that contains repetitive elements, genic elements, or neither. (c) Genome assembly length is strongly correlated to the total amount of repetitive DNA and weakly correlated with (d) the total gene length and (e) the number of genes. Phylogenetic tree names (a) are colored based on their diatom classification. The blue lines in (c–e) are the regression lines from the PGLS analyses.

*Psammoneis japonica* is a marine benthic diatom that forms straight or zigzag filaments (Figure 1a) and grows on sand grains. Although originally described from coastal waters in Japan (Sato et al., 2008), the strain sequenced here originated from Guam, expanding the known distribution of this species. *Psammoneis japonica* is a pennate diatom that lacks both a labiate process (rimoportula) and a raphe (Figure 1). The pennate diatom clade consists of a paraphyletic grade of raphe‐lacking species (araphid pennates) and a clade of raphe‐bearing species (raphid pennates; Figure 2a). Araphid pennates are undersampled in genomic studies, so the reference‐quality genome of *Psammoneis japonica* fills an important gap in our understanding of genome evolution in diatoms (Figure 2a).

### The nuclear genome of *Psammoneis japonica*

We generated roughly 1.2 million PacBio reads, yielding 8.6 Gb of raw data (Table S1). The N50 read length was 11.8 kb (Table S1). Illumina sequencing yielded 10.3 Gb of paired‐end DNA reads (Table S1). The nuclear genome of *Psammoneis japonica* totaled 91.4 Mb in length, which is roughly 50% larger than *Fragilaria crotonensis* (62 Mb; Figure 2b; Table 1), currently the only other reference‐quality genome published for an araphid diatom (Zepernick et al., 2022). The average sequencing depths of the final assembly using the PacBio and Illumina reads were 32× and 80×, respectively. After polishing the assembly with higher accuracy Illumina reads, the consensus quality value (QV) of the genome indicated >99.9% accuracy of the genome sequence. The final assembly contained 88% of stramenopile BUSCOs, which was similar to other high‐quality diatom genome assemblies (Table 1).

**TABLE 1:** General features of the *Psammoneis japonica* nuclear genome.

**Genome assembly**	
Estimated haploid genome size (Mb)	94.8
Assembly length (Mb)	91.4
Number of scaffolds	597
Scaffold N50 (kb)	378
GC content (%)	48.6
Repetitive DNA (%)	32.5
BUSCO completeness (single‐copy, duplicated) (stramenopiles_odb10)	88% [81%, 7%]
BUSCO completeness (single‐copy, duplicated) (eukaryota_odb10)	78% [73.3%, 4.7%]
**Genome annotation**	
Number of protein‐coding genes	11,047
Number of predicted proteins	11,119
Average gene length (bp)	1461
Average exon length (bp)	710
Predicted proteins with Pfam or PANTHER domain (%)	85.4
Predicted proteins with homology to SwissProt (%)	52
Predicted proteins with homology to UniProt Reference Proteomes (%)	88.5
BUSCO completeness of annotation (single‐copy, duplicated)	72% [66%, 6%]
OMArk completeness of annotation (single‐copy, duplicated)	76.39% [66.99%, 9.40%]

Approximately one third of the *Psammoneis japonica* genome is composed of repetitive DNA (Figure 2b; Table 1), which is comparable to similarly sized diatom genomes (Figure 2b; Table S2). A focused study of one diatom lineage, Thalassiosirales, revealed a strong correlation between genome size and repeat content (Roberts et al., 2024). Broadening this analysis to all currently available diatom genomes confirmed this trend, with larger genomes containing proportionally more repetitive DNA (PGLS, *F* = 198.2, *df* = 28, *p* < 0.001; Figure 2c; Table S3). In general, LTRs were the largest contributors to the total repeat content in *P. japonica*, as has been seen in other diatoms as well (Figure S1). A large fraction of the repeats could not be classified; however, among the different retroelement and transposon classes, only LTR abundance was correlated with genome size (PGLS, *F* = 21.14, *df* = 24, *p* < 0.001; Table S3).

After removal of incomplete gene models and single‐exon genes with no identifiable protein domains, the genome contained 11,047 protein‐coding genes (Table 1). Nearly 88% of gene models had putative functional annotations assigned based on matches to UniProt, Pfam, or PANTHER databases (Table 1). Across diatoms, genome size was weakly correlated with summed gene length (PGLS, *F* = 20.1, *df* = 28, *p* < 0.001; Figure 2d; Table S3) and total number of genes (PGLS, *F* = 13.2, *df* = 28, *p* < 0.01; Figure 2e; Table S3). The latter correlation, however, disappeared in a multivariate model that included total gene length, gene number, and repetitive DNA content together as predictors of genome size (PGLS, *F* = 96.2, *df* = 26, *p* = 0.21; Table S3). Dense genomic sampling in one diatom lineage, Thalassiosirales, also revealed a subordinate role for genic DNA in the evolution of genome size (Roberts et al., 2024). The importance of transposable elements, rather than genes, for explaining genome size variation in diatoms, mirrors patterns in many taxonomic groups and more broadly across eukaryotes (López‐Flores & Garrido‐Ramos, 2012).

### The microbiome of *Psammoneis japonica*

Sequencing of our xenic *Psammoneis japonica* culture recovered complete genomes from four co‐occurring bacterial species (Tables S4 and S5). All four genomes were classified as high quality based on the presence of all rRNA genes, ≥18 tRNAs, >90% completion, and <5% contamination (Bowers et al., 2017; Table S4). Phylogenetic placement of the four genomes—labeled 0F, 1F, 2F, and 3F—revealed a broad phylogenetic diversity of marine bacteria. The 0F genome was placed in the placeholder family UTPLA1 within the class Phycisphaerae (Planctomycetota) with an average nucleotide identity (ANI) of 76.2%. Phycisphaerae were originally described from a cultured *Porphyra*, a red algal seaweed that also grows in nearshore marine habitats (Fukunaga et al., 2009). The 1F genome was placed within the genus *Algihabitans*, a member of the placeholder family DSM‐21159 of the Alphaproteobacteria (ANI = 80.1%). *Algihabitans* was described from a cultured marine seaweed, the green algal species *Ulva prolifera*, isolated from offshore waters (Wang et al., 2019). The 2F genome was placed within *Ekhidna* (ANI = 78.2%), a genus of Bacteroidetes previously described from the South Pacific Gyre (Alain et al., 2010). Finally, the 3F genome was placed in *Balneola* (ANI = 77.2%), another genus of marine Bacteroidetes (Urios et al., 2006). Although the relatively low sequence similarity of these genomes to their closest references in existing databases emphasizes their novelty, metabarcode sequencing of additional diatom cultures from Guam determined members of *Balneola, Ekhidna*, and Phycisphaerae are among the most abundant members of the benthic bacterial community (Barreto Filho et al., 2021).

## CONCLUSIONS

As a chain‐forming inhabitant of the marine benthos, coupled with its phylogenetic position within a genomically uncharacterized lineage of araphid pennate diatoms, the reference genome of *Psammoneis japonica* fills several important gaps in genomic sequencing coverage across diatoms. As the number of sequenced diatom genomes continues to grow, the probability of capturing and characterizing the genomic basis of key transitions—such as the myriad traits associated with the evolution of araphid pennate and then raphid pennate diatoms (Nakov et al., 2018)—also increases. The *P. japonica* genome provides a valuable resource for comparative genomic studies, such as the construction of orthologous gene clusters between araphid and raphid pennate diatoms. The genome also verifies the accumulation of repetitive elements as the principal driver of genome size in diatoms, with lesser roles for gene number and length. The total bacterial diversity associated with diatoms is probably much greater in nature than it is in culture (Barreto Filho et al., 2021; Focardi et al., 2025; Stock et al., 2022), so although the full natural bacterial microbiome of *P. japonica* is unknown, the glimpse provided by these data revealed four novel species with only moderate similarity to known bacteria. This result supports the idea that bacterial species richness in the phycosphere is underdescribed and far exceeds the number of diatom host species, which themselves number in the tens to hundreds of thousands of species. Broadening the scope of diatom genome projects to include their bacterial symbionts is likely to reveal vast numbers of new species and help us understand why many diatom species cannot survive in isolation. The addition of these bacterial genomes will support further research into phycosphere metabolism, diatom–bacterial interactions, and biogeochemical cycles.

## AUTHOR CONTRIBUTIONS

**Wade R. Roberts:** Data curation (equal); formal analysis (equal); investigation (equal); methodology (equal); visualization (equal); writing – original draft (equal); writing – review and editing (equal). **Matthew Parks:** Conceptualization (equal); data curation (equal); formal analysis (equal); investigation (equal); methodology (equal); writing – review and editing (equal). **Marissa Ashner:** Formal analysis (equal); investigation (equal). **Matthew P. Ashworth:** Resources (equal); writing – review and editing (equal). **Nina Denne:** Formal analysis (equal); investigation (equal). **Elizabeth C. Ruck:** Methodology (equal); resources (equal); writing – review and editing (equal). **Elias Spiliotopoulos:** Formal analysis (equal); investigation (equal). **Anni Wang:** Formal analysis (equal); investigation (equal). **Shady A. Amin:** Conceptualization (equal); writing – review and editing (equal). **Sarah Schaack:** Conceptualization (equal); funding acquisition (equal); project administration (equal); resources (equal); supervision (equal); writing – review and editing (equal). **Norman J. Wickett:** Conceptualization (equal); funding acquisition (equal); project administration (equal); resources (equal); supervision (equal); writing – review and editing (equal). **Andrew J. Alverson:** Conceptualization (equal); funding acquisition (equal); project administration (equal); resources (equal); supervision (equal); writing – review and editing (equal).

## Supporting information


**Figure S1.** Proportions of total repeats per genome belonging to different repetitive element classes.

**Table S1.** Sequencing statistics.
**Table S2**. Diatom genome assembly data set for comparative analyses.
**Table S3**. Results of phylogenetic generalized least squares analyses.
**Table S4**. Assembly statistics for the metagenome‐assembled bacterial genomes.
**Table S5**. Number of uniquely mapped reads to the *Psammoneis japonica* nuclear genome, the four bacterial metagenome assembled genomes (MAGs), or neither.

## Data Availability

The sequencing data and assemblies are available from NCBI BioProject PRJNA476996. The nuclear, chloroplast, and mitochondrial genomes of *Psammoneis japonica* are available from GenBank accessions QPGO00000000, PX413109, and MG148339. The genome assemblies, gene models, annotations, and code to reproduce the figures are available from Zenodo (10.5281/zenodo.17247355).
