# Supplementary material for: Reference genome for the benthic marine diatom Psammoneis japonica: Bacterial associations and repeat‐driven genome size evolution in diatoms
Source: J Phycol. 2025 Nov 10;61(6):1556–65. doi: 10.1111/jpy.70101 (PMC12718438; doi:10.1111/jpy.70101)

**Figure S1.** Proportions of total repeats per genome belonging to different repetitive element classes.

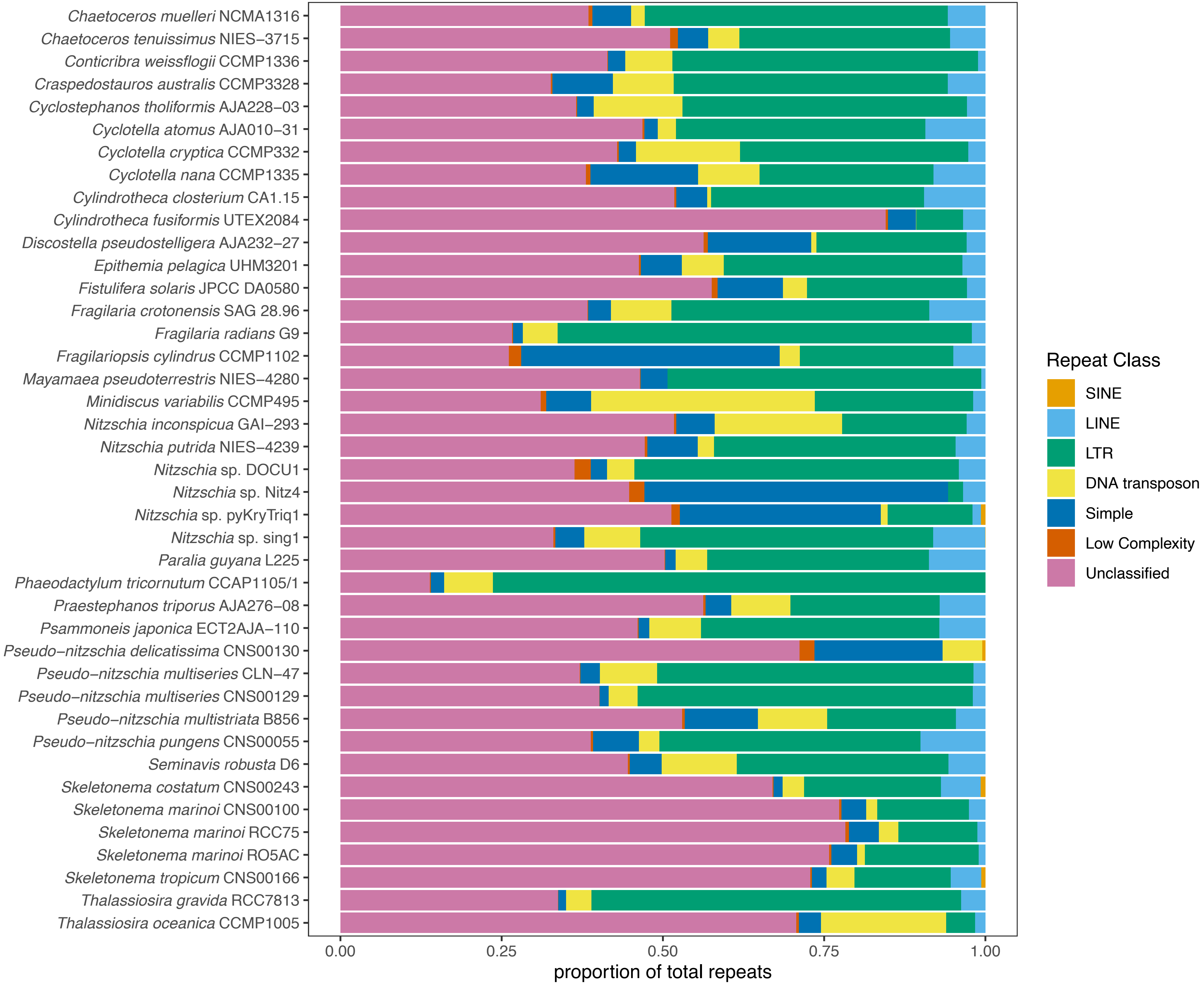

Supplement: Supplementary file 1 — Figure S1. Proportions of total repeats per genome belonging to different repetitive element classes. [file JPY-61-1556-s002.pdf]
